# Cardiovascular Effects of Whole-Body Cryotherapy in Non-professional Athletes

**DOI:** 10.3389/fcvm.2022.905790

**Published:** 2022-06-10

**Authors:** Francesca Coppi, Marcello Pinti, Valentina Selleri, Giada Zanini, Roberta D'Alisera, Pasqualino Maietta Latessa, Ferdinando Tripi, Gustavo Savino, Andrea Cossarizza, Milena Nasi, Anna Vittoria Mattioli

**Affiliations:** ^1^Department of Surgery, Medicine, Dentistry and Morphological Sciences, University of Modena and Reggio Emilia, Modena, Italy; ^2^Department of Life Sciences, University of Modena and Reggio Emilia, Modena, Italy; ^3^National Institute for Cardiovascular Research—INRC, Bologna, Italy; ^4^Department of Public Healthcare, Sport Medicine Service Azienda USL of Modena, Modena, Italy; ^5^Department of Quality of Life, “Alma Mater Studiorum”, Bologna, Italy; ^6^“La Fratellanza 1874” Not-for-profit sport Association, Modena, Italy; ^7^Department of Medical and Surgical Sciences for Children and Adults, University of Modena and Reggio Emilia, Modena, Italy

**Keywords:** cardiovascular systems, vital signs, heart rate, respiratory rate, oxygen saturation, whole-body cryotherapy, blood pressure

## Abstract

**Objectives:**

The study aimed to investigate changes in heart rate, blood pressure, respiratory rate, oxygen saturation, and body temperature in non-professional trained runners during whole body cryotherapy (WBC).

**Methods:**

Ten middle-distance runners received 3 once-a-day sessions of WBC. Subjects underwent BP measurements and ECG recorded before and immediately after the daily WBC session. During WBC we recorded a single lead trace (D1) for heart rhythm control. In addition, the 5 vital signs Blood pressure, heart rate, respiratory rate, oxygen saturation, and body temperature were monitored before, during, and after all WBC session.

**Results:**

We did not report significant changes in ECG main intervals (PR, QT, and QTc). Mean heart rate changed from 50.98 ± 4.43 bpm (before) to 56.83 ± 4.26 bpm after WBC session (*p* < 0.05). The mean systolic blood pressure did not change significantly during and after WBC [b baseline: 118 ± 5 mmHg, changed to 120 ± 3 mmHg during WBC, and to 121 ± 2 mmHg after session (*p* < 0.05 vs. baseline)]. Mean respiratory rate did not change during WBC as well as oxygen saturations (98 vs. 99%). Body temperature was slightly increased after WBC, however it remains within physiological values

**Conclusion:**

In non-professional athletes WBC did not affect cardiovascular response and can be safely used. However, further studies are required to confirm these promising results of safety in elderly non-athlete subjects.

## Introduction

Whole body cryotherapy (WBC), short exposure to dry air at cryogenic temperatures, has recently been applied to recovery after injury during sports and to counteract the inflammatory response due to specific diseases, characterised by high levels of inflammation such as rheumatoid arthritis (1–3).

The use of cryotherapy as an alternative or in association with immersion in cold water is widespread among professional athletes and is also spreading among non-professional athletes. However, the latter carry out the treatment WBC without medical supervision. Consequently, it is important to know the effects of cold temperature on cardiovascular parameters, especially on blood pressure in order to define the level of safety of the procedure. WCR results in rapid and especially short-term exposure to low temperatures.

Recent reports suggest that one or more WBC sessions can induce anti-inflammatory effects (4). In a previous study, we analysed a large panel of cytokines, haematological, and hormonal parameters, as well as circulating mitochondrial DNA (1, 5). We found that in non-professional athletes, WBC induced beneficial immunological and metabolic responses and seems to promote tissue repair (1).

To our knowledge little is known about the effect of cryogenic temperature during WBC on cardiovascular parameters.

Using state of the art technologies, we investigated changes in heart rate, blood pressure, respiratory rate, and oxygen saturation in non-professional trained runners. In addition, we also evaluated changes in electrocardiogram immediately after WBC and after acute exposure.

## Methods

### Subjects

Ten volunteer middle-distance non-professional runners were recruited by dedicated sport clubs for consecutive, 2-min WBC sessions, proposed over a 3-day schedule. The mean age was 38 ± 12 years. Subjects with known injuries or inflammatory diseases were excluded. During the study, the athletes' training programs were maintained from previous weeks: specifically, runners trained once a day (average 60 min/run). We selected a group of runners belonging to the same non-professional group who were used to training together. The training of runners was scheduled for alternating a day of 20-min run with an easy warm-up plus aerobic repeats and a day of 40/70 min of endurance running. No change in the running program was performed during the study. Training sessions were in the evenings. WBC sessions (as well as blood and urine collection) were conducted at lunchtime.

Lifestyle and cardiovascular risk factors were investigated. Specifically cardiovascular risk factors included: familiarity, blood lipid levels, smoking, hypertension, diabetes, body mass index. Caffeine consumption was evaluated: we investigated frequency and quantity of coffee (espresso coffee, cappuccino), tea and caffeinated drinks (including energy drinks and sports drinks).

The local Ethics Committee approved the study (Area Vasta Nord Emilia Romagna #88/2018/SPER/AUSLMO) and each subject provided written informed consent. Volunteers were involved in the reporting and dissemination of our research.

### ECG

Subjects underwent BP measurements and ECG recorded before and immediately after the daily WBC session. During WBC we recorded a single lead trace (D1) for heart rhythm control. The following parameters were measured and compared before and after WBC session: heart rate (bpm), rhythm, QT and QTc intervals (msec), PR interval (msec), number of supraventricular ectopic beats (SE), number of ventricular ectopic beats (VB).

The study's workflow has been previously published (1).

### Vital Signs and Cardiovascular Parameters

Non-invasive hemodynamic assessment will be determined by recording the 5 WHO recommended vital signs through electrocardiography and photo plethysmography signals, acquired from the subjects' hands, carefully calibrates the data according to mathematical models. ButterfLife^®^ is a non-invasive patented medical device that records the 5 vital signs [heart rate (bpm), blood pressure (mmHg), respiratory rate (rpm), oxygen saturation (%), and body temperature (°Celsius)] simultaneously and provides an output of behaviour of the signs that can be easily analysed in real time or by remote clinical staff in telemedicine model. Every 5 s the software emits the 5 vital parameters in addition to the signals visible on the ECG monitor and photo plethysmography.

Thanks to the integrated artificial intelligence, the system acquires and sends data every 5 s and creates a trend of the parameters, allowing a reliable and customizable detection of the parameters over time, comparable with the previously recorded health data. An Integrated artificial intelligence algorithm elaborates parameter of Heart Rate Variability (HRV). The time-domain parameters of HRV evaluated were SD of RR intervals, root mean- squared difference in successive RR intervals, and percentage of successive RR intervals 50 ms different from one another. The frequency domain parameter of HRV evaluated will be high-frequency power (0.15 to 0.40 Hz). Sympathovagal balance will be assessed by low-frequency power/high-frequency power ratio. Vital signs recording allows for a simple assessment of the patient's underlying health conditions in order to proceed with safe and tailored exercise. It also allows the monitoring of clinical conditions during the WBC and changes of the hemodynamic function (6, 7).

### Statistical Analysis

Quantitative variables were compared between pre- and post-WBC by the Wilcoxon matched pairs signed rank test or by two-way ANOVA and Sidak's multiple comparisons test. Correlations between values recorded with traditional methods and recorded with BF were explored with linear regression analysis. Multivariate analysis was performed to exclude potential confounding factors. Due to the correlation between heart rate and blood pressure these variables were analysed separately.

Potential confounders included cardiovascular risk factors and lifestyle habits, i.e., smoking habits, intake of energy drinks, and caffeinated beverages.

*P*-values < 0.05 were considered statistically significant. Statistical analyses were performed using Prism 6.0 (Graphpad Software Inc., La Jolla, USA).

## Results

Clinical characteristics and blood analytical chemistry parameters of subjects are shown in Table 1. No change in the running program was performed during the study. Subjects were asymptomatic during all WBC sessions.

**Table 1:** Baseline characteristics of athletes.

**Nr of subjects**	**10**
Mean age (years)	38 ± 12
Mean BMI (kg/m2)	19.5 ± 2
**Cardiovascular risk factors**	
Hypertension nr of subjects and (%)	0 (0)
Type II Diabetes nr of subjects and (%)	0 (0)
Hypercholesterolemia nr of subjects and (%)	1 (10)
Smoking habitus (active or previous) (nr of subjects and (%)	7 (70)
Obesity (BMI > 30 Kg/m^2^)	0 (0)
Energy drinks consumption (nr of subjects and (%)	4 (40)
Habitual coffee consumption	
(nr of subjects and (%)	6 (60)
Cardiovascular drugs	0 (0)
Beta-blockers	0 (0)
Lowering lipid drugs	0 (0)
**Blood analytical chemistry**	
Glucose (mg/dL)	80.60 ± 12.66
Urea (mg/dL)	35.40 ± 6.15
Creatinine (mg/dL)	0.90 ± 0.10
Cholesterol (mg/dl)	205.80 ± 51.85
HDL cholesterol (mg/dl)	66.80 ± 13.38
LDL (mg/dl)	125.40 ± 38.52
Triglycerides (mg/dl)	100.00 ± 43.52
GOT (U/L)	28.20 ± 8.11
GPT (U/L)	23.90 ± 13.49
GGT (U/L)	26.90 ± 21.28
CK (U/L)	262.30 ± 157.20
Amylase (U/L)	84.70 ± 21.03
Sodium (mEq/L)	139.50 ± 1.08
Potassium	4.30 ± 0.38
Iron (mEq/L)	100.70 ± 40.54
**Cardiovascular vital signs**	
Heart rate (bpm)	50.98 ± 4.43
Systolic Blood Pressure (mmHg)	118 ± 6
Diastolic blood pressure (mmHg)	76 ± 2
Respiratory rate (rpm)	14.5 ± 2
Oxygen saturation (%)	98%

*HDL, high-density lipoprotein*.

### Changes in ECG Parameters

ECG was recorded before and after WBC session. We did not report significant changes in ECG main intervals (PR, QT, and QTc). Mean heart rate changed from 50.98 ± 4.43 bpm (before) to 56.83 ± 4.26 bpm after WBC session (*p* < 0.05). During WBC we recorded rare supraventricular ectopic beats in 2 subjects. No ventricular ectopies were registered during WBC sessions.

### Changes in Vital Signals Parameters

**Figure 1:**
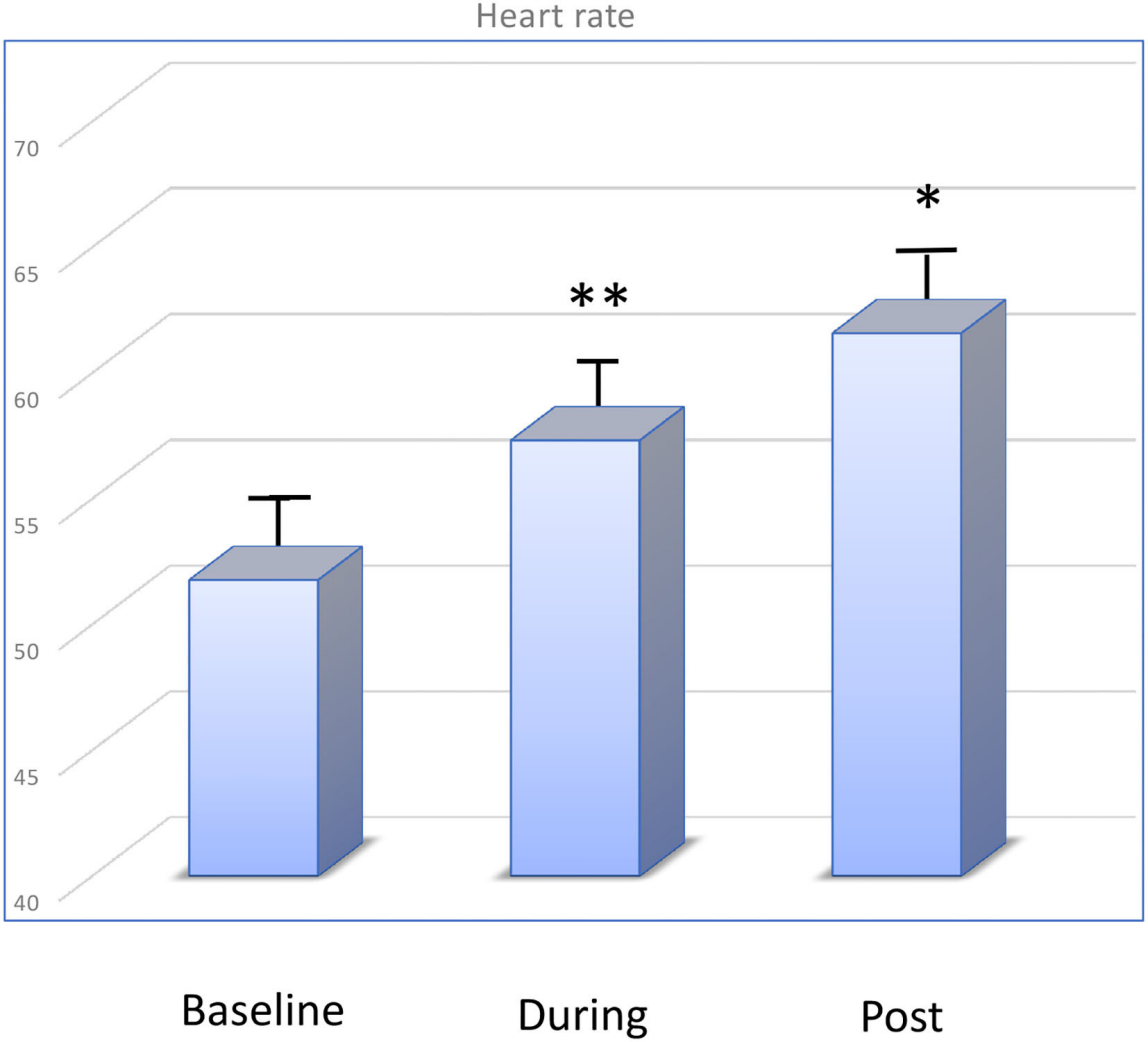
Changes in heart rate (bpm) at baseline, during, and after WBC.

The mean heart rate before WBC was 51.75 ± 4.56 bpm, changed to 57.23 ± 5.12 bpm during WBC (*p* < 0.01) and to 61.5 ± 3.67 bpm after session (*p* < 0.05). The trend was an increase of the heart rate during exposure to very low temperature, however values remain within normal range. This trend reduced from the first to the last sessions (Figure 1).

**Figure 2:**
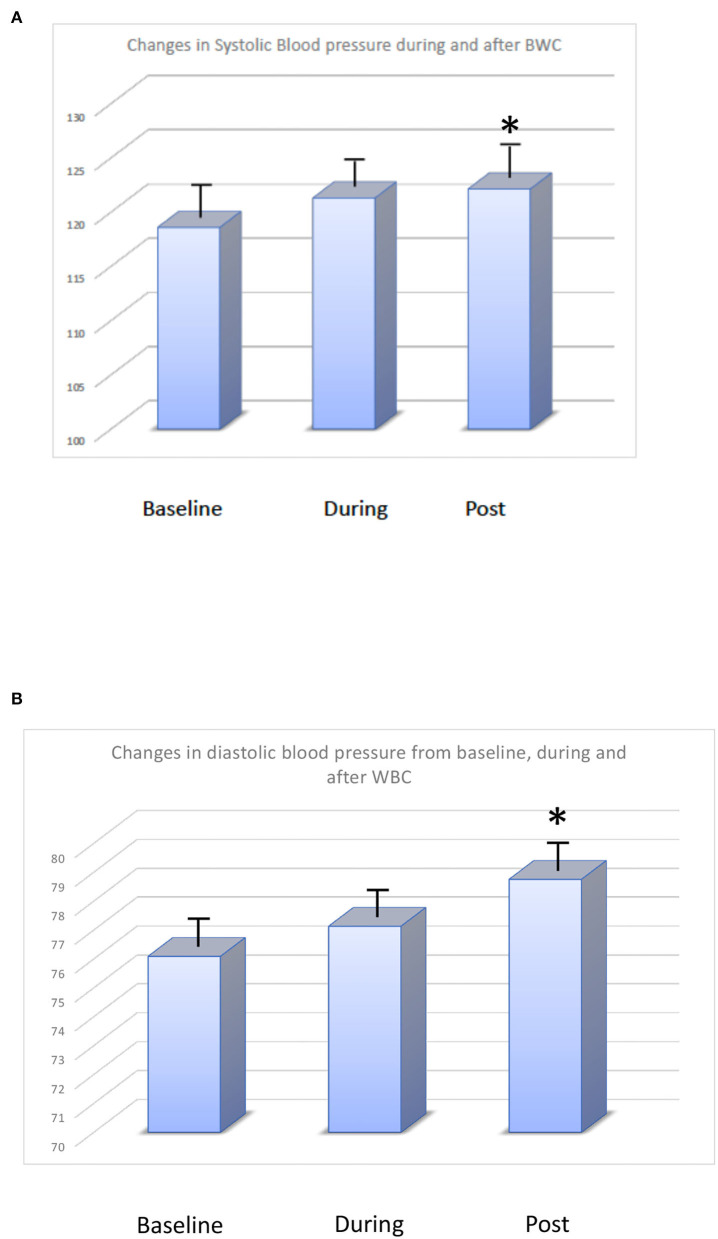
**(A)** Changes in systolic blood pressure (mmHg) at baseline, during, and after WBC. **(B)** Changes in diastolic blood pressure (mmHg) at baseline, during, and after WBC.

The mean systolic blood pressure did not change significantly during and after WBC (baseline: 118 ± 5 mmHg, changed to 120 ± 3 mmHg during WBC and to 121 ± 2 mmHg after session (*p*< 0.05 vs. baseline). Figure 2 shows changes in systolic and diastolic blood pressure during the WBC sessions. All subjects showed an increase of values after WBC however changes were small and values remained within normal range.

**Figure 3:**
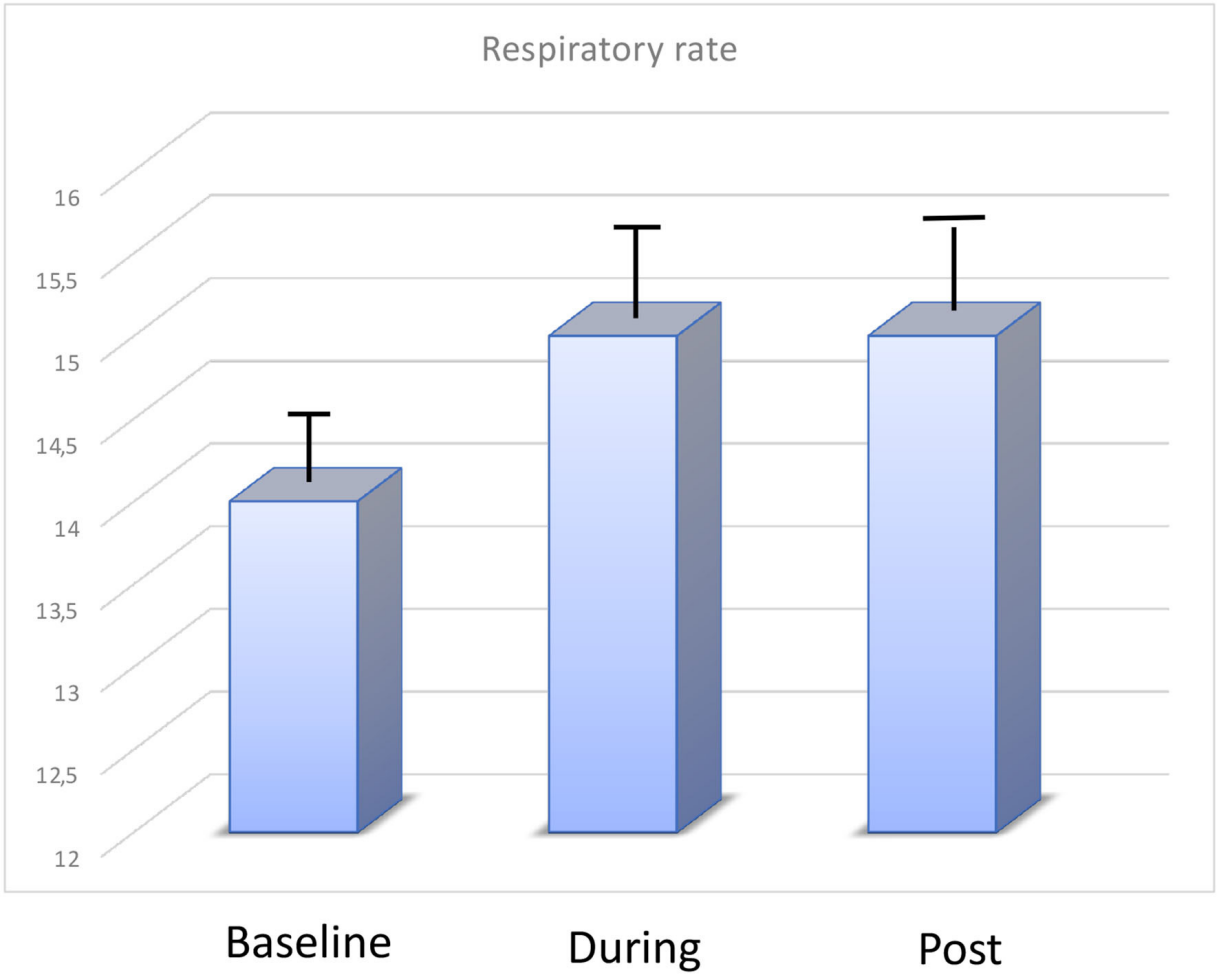
Changes in respiratory rate (rpm) at baseline, during, and after WBC.

Mean respiratory rate did not change during WBC (before 14.5 ± 2 rpm changed to 15 ± 1 rpm) as well as oxygen saturations (98% vs. 99%) (Figure 3).

Body temperature was slightly increased after WBC; however, it remains within physiological values. After the completion of the WBC treatments, the skin temperature normalised very quickly.

Due to the small number of subjects included in the study and the homogeneous characteristics of population we could only include smoking habits and the intake of energy drinks and caffeinated drinks as confounding factors. We found no significant differences in results after analysing the results according to these variables.

## Discussion

The current study evaluates the cardiovascular effects of WBC treatment in non-professional male runners. The main feature of the present study was that WBC did not change cardiovascular parameters.

WBC has been shown to be very effective on human health especially in its action on inflammation and muscle damage related to high exercise. The effects on inflammatory biomarkers (IL-6, IL 9 etc) are reported in several studies as well as the effects on the lipid and hormonal structure (1–3). The use of cold to reduce the inflammatory response triggered by strenuous physical activity is widespread among high intensity professional and non-professional athletes (1, 8). According to that reported in previous studies, WBC had an overall beneficial effect of on lipid, glucose, and protein metabolism (1). The effects on lipid levels were reported after at least 10 WBC sessions. In a previous study, we found an improvement of HDL after 3 sessions of WBC suggesting a sub-acute effect (9, 10). WBC also acts on skeletal muscle contraction and fatigue (11, 12). Furthermore, one of the most frequent complications in athletes, especially in the elderly, are cramps. Muscle cramp is a sudden, severe, and involuntary muscle contraction or excessive shortening. Symptoms range from mild to severe pain to paralysis-like immobility (13).

Several hypotheses have been advanced to explain the origin of muscle cramp induced by exercise, i.e., dehydration and electrolyte depletion hypothesis and neuromuscular hypothesis (13). In both cases a trigger for muscle cramps is high-humidity environment and elevated heat conditions. Although there is no evidence that WBC has beneficial effects on cramps, the climatic conditions generated by exposure to cold could counteract the appearance of exercise-induced cramps. This hypothesis is at the moment theoretical and to be proved but certainly compelling.

Little is known about the cardiovascular effects of WBC. Previous studies have reported an increased risk of myocardial infarction, stroke, and blood pressure when the external temperature decreases (14–16). The mechanisms proposed to explain this association are systemic inflammation, thrombosis, and vasoconstriction (17). However, most of the studies refer to the external temperature; on the contrary, the evidence on the cardiovascular effects of rapid exposure to cold is scarce. BMI and fat could influence cardiovascular effects induced by exposure to cold temperature. Young runners usually have a low BMI with high muscle and low fat. However, BMI is not a good measure for athletes because it does not allow evaluating differences in lean body mass and fat mass. Conventional Bioelectrical Impedance Analysis or Bioelectrical Impedance Vector Analysis can give more reliable details about body composition differences in competitive and non-competitive subjects, outlining a progressive decline in ECW and increase in ICW without affecting TBW composition of athletes (18, 19).

Nonetheless, exposure to cold through WBC is a habit that is spreading for selective reduction of adipose tissue, anti-ageing, and as a release of stress (20–23).

Wiecek and colleagues found that repeated whole-body exposure to cryogenic temperature increased inducible nitric oxide synthase (iNOS) concentration in senior subjects, regardless of their physical activity level (22).

We evaluated cardiovascular safety of WBC through measurements of vital signs during sessions. The new patented and certified system we used allow us to get data during all treatment exposure. The average and standard deviation of vital parameters calculated for both the ButterfLife and the reference methods show an accurate and repeatable accordance between the devices (Data showed in Supplemental Materials). Systolic, diastolic, and mean blood pressure showed a small change during WBC and these changes attenuates during repetitive exposure supporting the hypothesis that the first value recorded during the first exposure are induced by sympathetic activation. Similarly, heart rate and respiratory rate showed small changes during cold exposure and come back to previous values quickly. Oxygen saturation did not change during WBC. All these data suggest that WBC can be used safely in young athletes.

Limitation of the study. The main limitation of the study is the low number of subjects included in the study. The selection of patient populations was very strict to avoid bias, as previously stated the runners belong to the same non-professional group who were used to training together. A second limitation is the young age of the study subjects, which makes it difficult to transfer the results to the population of older non-professional athletes.

However, from a practical clinical point of view, the knowledge of the effects of acute cold on cardiovascular parameters is important to prevent unexpected and dangerous events, especially due to the widespread diffusion of physical activity and sports even in non-professional subjects. WHO guidelines suggest increased physical activity as a way to prevent non- communicable diseases, but when it comes to adults and elderly, it is important to expand our knowledge on safety (24–28).

Further investigations are needed to evaluate its safety in elderly non-professional athletes and also in elderly non-athletes.

## Conclusions

As a whole, a main conclusion can be drawn from the present study. In non-professional athletes WBC did not affect cardiovascular response and may be a protagonist in promoting a safety process of tissue repair. However, further studies are required to confirm these promising results of safety in elderly non-professional athletes and also in elderly non-athlete subjects.

## Interest in the field

Whole body cryotherapy (WBC) seems to have a beneficial effect on tissue repair, innate, and adaptive immune systems in non-professional male athletes. In recent years, WBC has also spread to non-professional athletes and adult/elderly subjects for its anti-inflammatory and anti-aging effects. It becomes essential to verify the safety of this method especially at the cardiovascular level.

## Data Availability Statement

The raw data supporting the conclusions of this article will be made available by the authors, without undue reservation.

## Ethics Statement

The studies involving human participants were reviewed and approved by (Area Vasta Nord Emilia Romagna #88/2018/SPER/AUSLMO). The patients/participants provided their written informed consent to participate in this study.

## Author Contributions

FC, MN, AC, AM, VS, and GZ contributed to the conception and the design of the work. MN, AM, and PL contributed to the draught and the final approval of the manuscript. FC, MN, AC, and AM contributed to the interpretation of data. RD'A, PL, GS, and FT enrolled the subjects for the study. All authors contributed to manuscript revision, read, and approved the submitted version.

## Funding

This work was supported by the Italian Ministry of Health, Section of Prevention of addictions, doping, and mental health (reference number Project 2017-1 Effects of whole body cryotherapy on inflammatory mechanisms and on the hormonal profile of athletes) and by University of Modena and Reggio Emilia Fund FAR Dipartimento CHIMOMO FAR 2022-1 (Immunological and cardiovascular effects of systemic cryosauna in menopausal women: focus on ageing).

## Conflict of Interest

The authors declare that the research was conducted in the absence of any commercial or financial relationships that could be construed as a potential conflict of interest.

## Publisher's Note

All claims expressed in this article are solely those of the authors and do not necessarily represent those of their affiliated organizations, or those of the publisher, the editors and the reviewers. Any product that may be evaluated in this article, or claim that may be made by its manufacturer, is not guaranteed or endorsed by the publisher.

## References

[1] Nasi M Bianchini E Lo Tartaro D De Biasi S Mattioli M Paolini A. Effects of whole-body cryotherapy on the innate and adaptive immune response in cyclists and runners. Immunol Res. (2020) 68:422–35. 10.1007/s12026-020-09165-1 33159311

[2] Peake JM Neubauer O Della Gatta PA et al. Muscle damage and inflammation during recovery from exercise. J Appl Physiol. (2017) 122:559–70. 28035017 10.1152/japplphysiol.00971.2016

[3] Patel K Bakshi N Freehill MT Awan TM. Whole-Body cryotherapy in sports medicine. Curr Sports Med Rep. (2019) 18:136–40. 10.1249/JSR.0000000000000584 30969239

[4] Partridge EM J Cooke A McKune DB Pyne. Whole-Body cryotherapy: potential to enhance athlete preparation for competition? Front Physiol. (2019) 10:1007. 10.3389/fphys.2019.01007 31447697 PMC6691163

[5] Zanini G De Gaetano A Selleri V Savino G Cossarizza A Pinti M. Mitochondrial DNA and exercise: implications for health and injuries in sports. Cells. (2021) 10:2575. 10.3390/cells10102575 34685555 PMC8533813

[6] Mattioli AV Ballerini Puviani M Malagoli A. Quarantine and isolation during COVID-19 outbreak: a case of online diagnosis of supraventricular arrhythmia through telemedicine. J Arrhythm. (2020) 36:1114–6. 10.1002/joa3.12431 33335637 PMC7733570

[7] Maffei F Nasi M. Vittori LN Bragonzoni L Toselli S Tripi F Mattioli AV Maietta-Latessa P. Adherence to an adapted physical activity program in sedentary adults. J Human Sport Exer. (2020) (1):1–12. 10.14198/JHSE.2022.171.05

[8] Lombardi G. Ziemann E. Banfi G. Whole-Body cryotherapy in athletes: from therapy to stimulation. an updated review of the literature. Front Physiol. (2017) 8:258. 10.3389/fphys.2017.00258 28512432 PMC5411446

[9] Wiecek M. Wiecek M Szymura J Sproull J Szygula Z. Decreased blood asprosin in hyperglycemic menopausal women as a result of whole-body cryotherapy regardless of metabolic syndrome. J Clin Med. (2019) 8:1428. 10.3390/jcm8091428 31510055 PMC6780623

[10] Lubkowska A Szygula Z Klimek AJ Torii M. Do sessions of cryostimulation have influence on white blood cell count, level of IL6 and total oxidative and antioxidative status in healthy men? Eur J Appl Physiol. (2010) 109:67–72. 10.1007/s00421-009-1207-2 19779735

[11] llen DG Westerblad H. Role of phosphate and calcium stores in muscle fatigue. J Physiol. (2001) 536:657–65. 10.1111/j.1469-7793.2001.t01-1-00657.x 11691862 PMC2278904

[12] Chen YY Kao TW Chou CW Wu CJ Yang HF Lai CH. Exploring the link between serum phosphate levels and low muscle strength, dynapenia, and sarcopenia. Sci Rep. (2018) 8:3573. 10.1038/s41598-018-21784-1 29476104 PMC5824959

[13] Giuriato G Pedrinolla A Schena F Venturelli M. Muscle cramps: a comparison of the two-leading hypothesis. J Electromyogr Kinesiol. (2018) 41:89–95. 10.1016/j.jelekin.2018.05.006 29857264

[14] Bhaskaran K Hajat S Haines A Herrett E Wilkinson P Smeeth L. Short term effects of temperature on risk of myocardial infarction in England and Wales: time series regression analysis of the Myocardial Ischaemia National Audit Project (MINAP) registry. BMJ. (2010) 341:c3823. 10.1136/bmj.c3823 20699305 PMC2919679

[15] Fustinoni O Saposnik G. Esnaola y Rojas MM, Lakkis SG, Sposato LA; ReNACer Investigators. Higher frequency of atrial fibrillation linked to colder seasons and air temperature on the day of ischemic stroke onset. J Stroke Cerebrovasc Dis. (2013) 22:476–81. 10.1016/j.jstrokecerebrovasdis.2013.03.009 23562211

[16] Jansen PM Leineweber MJ Thien T. The effect of a change in ambient temperature on blood pressure in normotensives. J Hum Hypertens. (2001) (2):113–7. 11317190 10.1038/sj.jhh.1001134

[17] Cai J Meng X Wang C Chen R Zhou J Xu X. The cold effects on circulatory inflammation, thrombosis and vasoconstriction in type 2 diabetic patients. Sci Total Environ. (2016) 568:271–77, doi.org/10.1016/j.scitotenv.2016.06.030. 27295598 10.1016/j.scitotenv.2016.06.030

[18] Meleleo D Bartolomeo N Cassano L Nitti A Susca G Mastrototaro G. Evaluation of body composition with bioimpedence. a comparison between athletic and non-athletic children. Eur J Sport Sci. (2017) 17:710–9. 10.1080/17461391.2017.1291750 28319679

[19] Mattioli AV. Coffee consumption effects on bioelectrical impedance parameters: does gender matter? Eur J Clin Nutr. (2022). 10.1038/s41430-022-01136-z [Epub ahead of print]. 35388165

[20] Suvaddhana Loap S SidAhmed-Mezi M Meningaud JP Hersant B. A prospective, comparative study (before and after) for the evaluation of cryothermogenesis' efficacy in body contouring: abdomen and saddlebags. Plast Reconstr Surg. (2022) 149:424e−428. 10.1097/PRS.0000000000008857 35196676

[21] Pilch W Wyrostek J Piotrowska A Czerwińska-Ledwig O Zuziak R Sadowska-Krepa E. Blood pro-oxidant/antioxidant balance in young men with class II obesity after 20 sessions of whole body cryostimulation: a preliminary study. Redox Rep. (2021) 26:10–7. 10.1080/13510002.2021.1881328 33560197 PMC7891890

[22] Wiecek M Szygula Z Gradek J Kusmierczyk J Szymura J. Whole-Body cryotherapy increases the activity of nitric oxide synthase in older men. Biomolecules. (2021) 11:1041. 10.3390/biom11071041 34356664 PMC8301999

[23] Doets JJR Topper M Nugter AM. A systematic review and meta-analysis of the effect of whole body cryotherapy on mental health problems. Complement Ther Med. (2021) 63:102783. 10.1016/j.ctim.2021.102783 34655758

[24] WHO guideline “*Stay physically active during self quarantine*”. Available online at: http://www.euro.who.int/en/health-topics/health-emergencies/coronavirus-covid-19/novel-coronavirus-2019-ncov-technical-guidance/stay-physically-active-during-~self-quarantine (accessed May 16, 2022).

[25] Nasi M Patrizi G Pizzi C Landolfo M Boriani G Dei Cas A et al. The role of physical activity in individuals with cardiovascular risk factors: an opinion paper from Italian society of cardiology-emilia romagna-marche and SIC-sport. J Cardiovasc Med. (2019) 20:631–9. 10.2459/JCM.0000000000000855 31436678

[26] Ricci F Izzicupo P Moscucci F Sciomer S Maffei S Di Baldassarre A. Recommendations for physical inactivity and sedentary behavior during the coronavirus disease (COVID-19) pandemic. Front Public Health. (2020) 8:199. 10.3389/fpubh.2020.00199 32574294 PMC7235318

[27] Amann M Venturelli M Ives SJ Morgan DE Gmelch B Witman MA. Group III/IV muscle afferents impair limb blood in patients with chronic heart failure. Int J Cardiol. (2014) 174:368–75. 10.1016/j.ijcard.2014.04.157 24794967 PMC4075285

[28] Paneroni M Pasini E Comini L Vitacca M Schena F Scalvini S. Skeletal muscle myopathy in heart failure: the role of ejection fraction. Curr Cardiol Rep. (2018) 20:116. 10.1007/s11886-018-1056-x 30259199

