# Supplementary material for: Cardiovascular Effects of Whole-Body Cryotherapy in Non-professional Athletes
Source: Front Cardiovasc Med. 2022 Jun 10;9:905790. doi: 10.3389/fcvm.2022.905790 (PMC9227663; doi:10.3389/fcvm.2022.905790)
Supplement: Supplementary file 2 [file Data_Sheet_2.pdf]

Figure
Supplemental materials
Systolic and diastolic blood pressure (mmHg) before, during and after WBC recorded with ECG and BF: comparison between traditional method (blue line) and BF (red line)

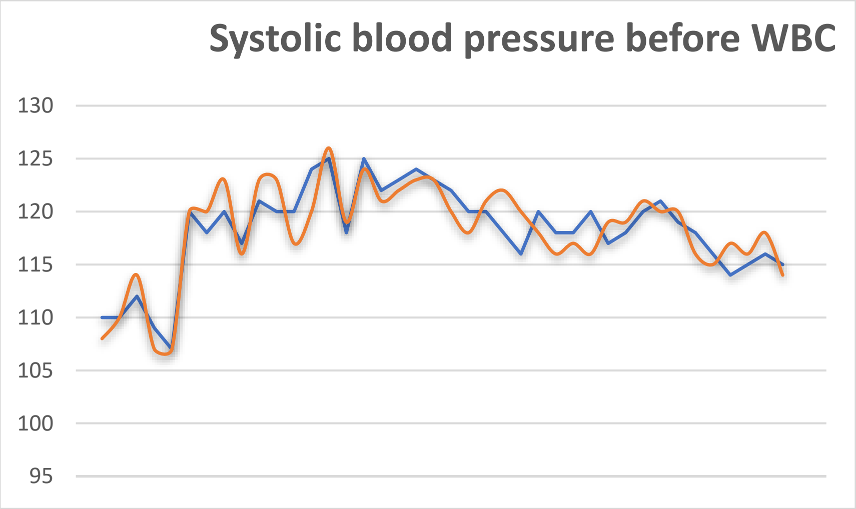

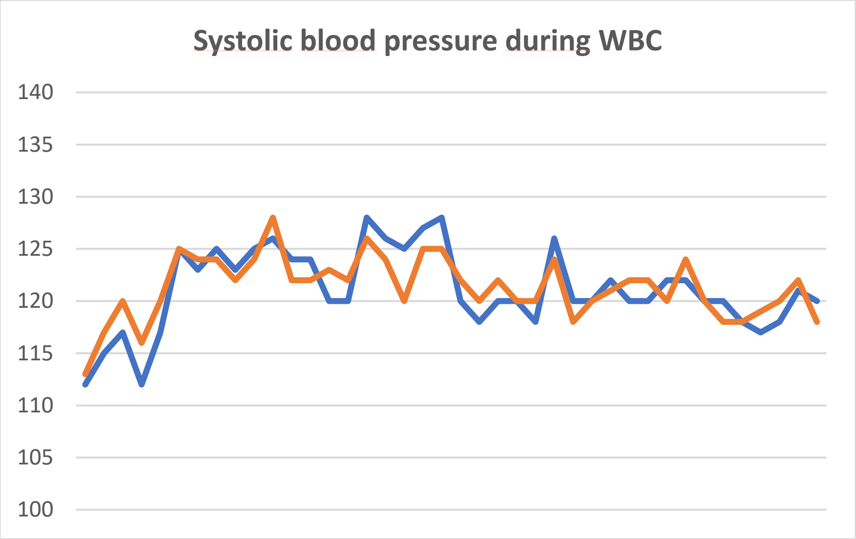

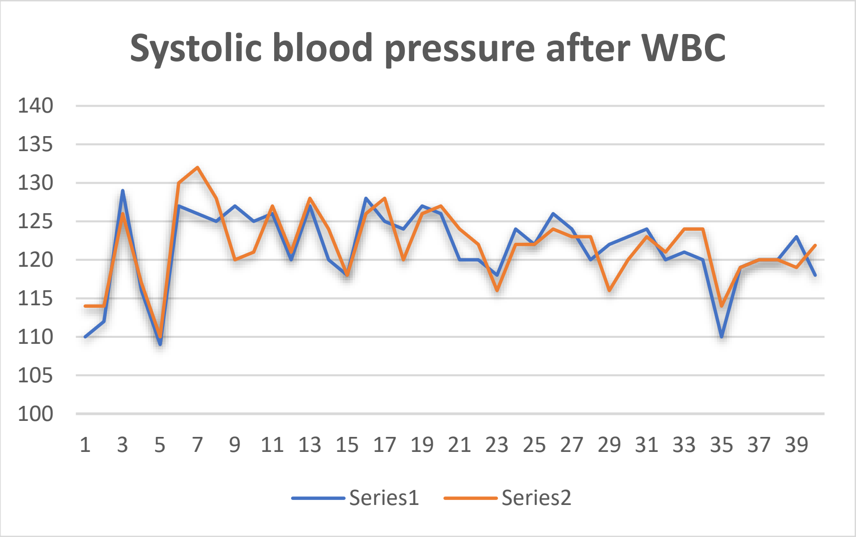

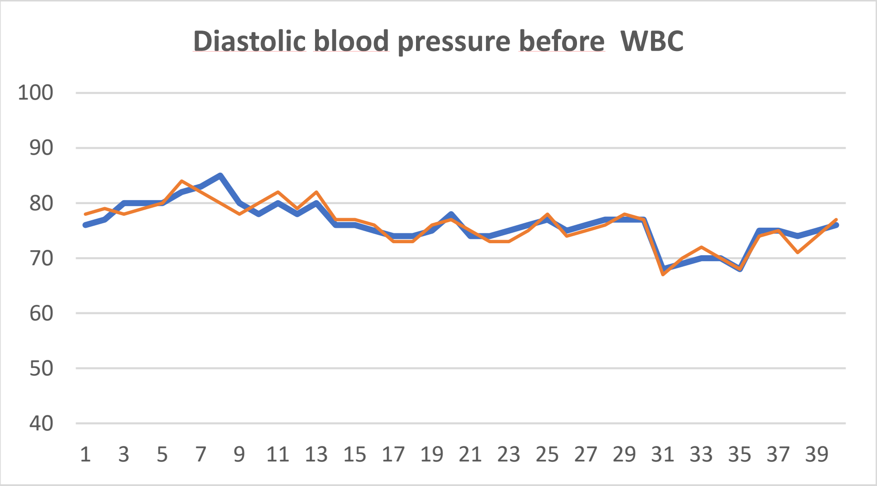
Diastolic blood pressure before WBC
100
90
80
70
60
50
40
1 3 5 7 9 11 13 15 17 19 21 23 25 27 29 31 33 35 37 39

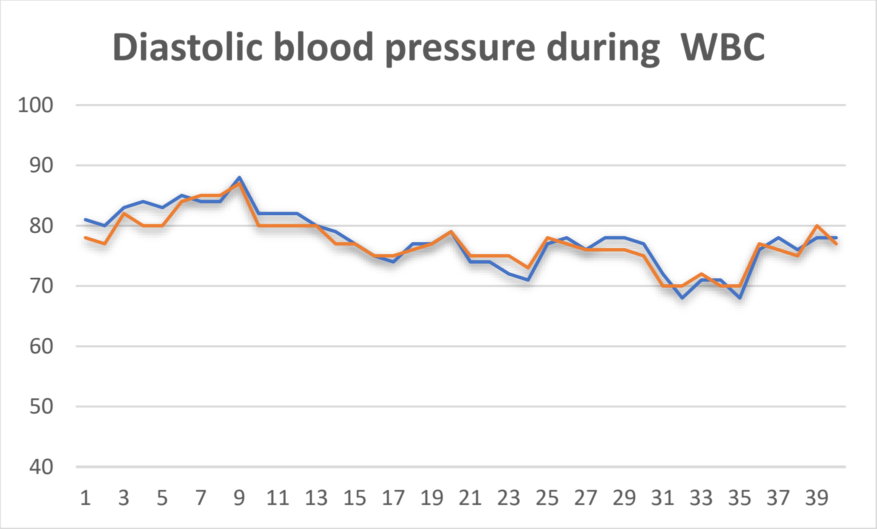
Diastolic blood pressure during WBC
100
90
80
70
60
50
40
1 3 5 7 9 11 13 15 17 19 21 23 25 27 29 31 33 35 37 39

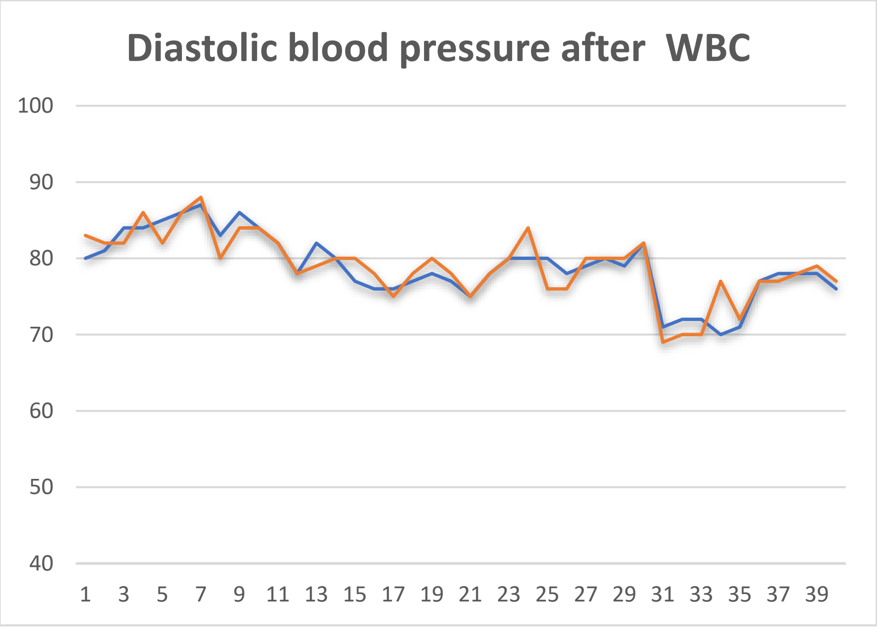
Diastolic blood pressure after WBC
100
90
80
70
60
50
40
1 3 5 7 9 11 13 15 17 19 21 23 25 27 29 31 33 35 37 39
