# Supplementary material for: Cardiovascular Effects of Whole-Body Cryotherapy in Non-professional Athletes
Source: Front Cardiovasc Med. 2022 Jun 10;9:905790. doi: 10.3389/fcvm.2022.905790 (PMC9227663; doi:10.3389/fcvm.2022.905790)

Figure
Supplemental materials
Respiratory rate (rpm) before and after WBC recorded with ECG and BF: comparison between traditional method (blue line) and BF (red line)

Before WBC

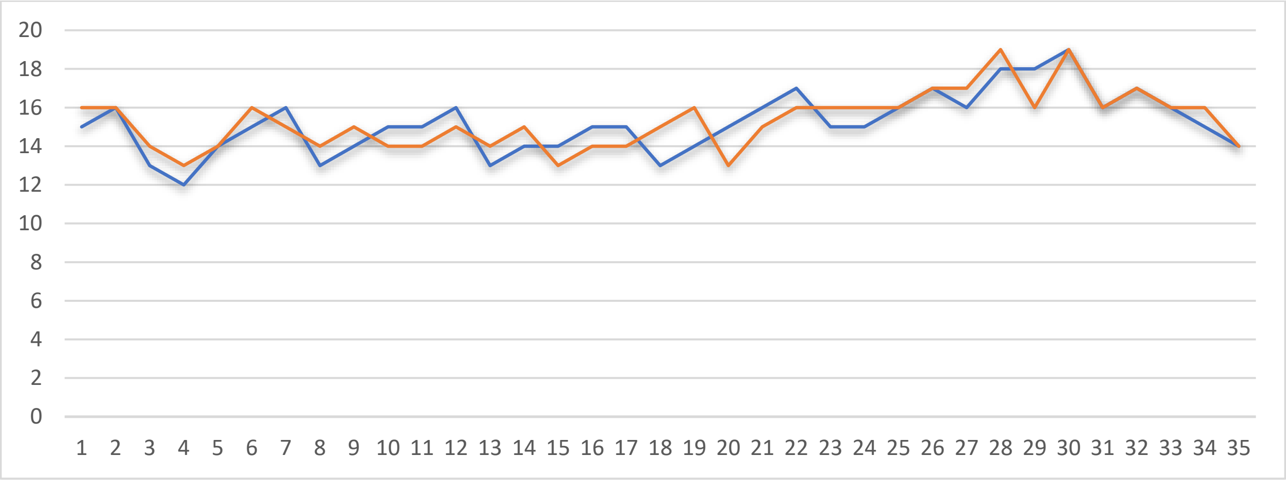

After

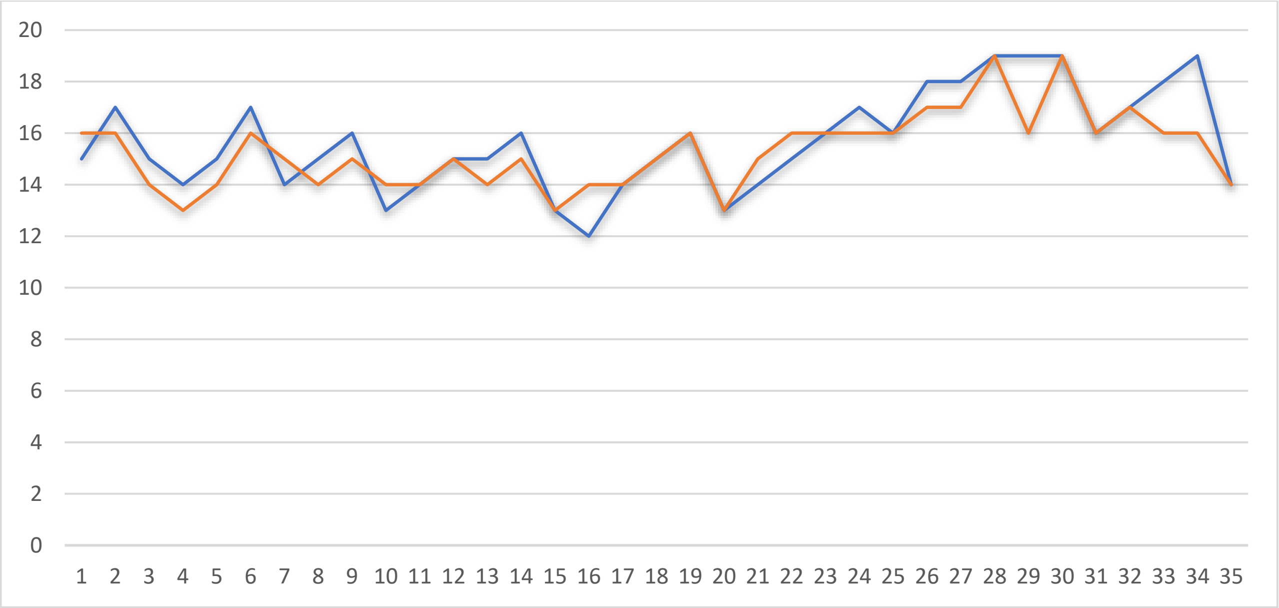

Supplement: Supplementary file 3 [file Data_Sheet_3.pdf]
